# Citrullinated peptides as drug candidates for rheumatoid arthritis

**DOI:** 10.3389/fimmu.2025.1648913

**Published:** 2025-11-25

**Authors:** Adrian H. Bustos, Mads Brüner, Tue Wenzel Kragstrup, Kira Astakhova

**Affiliations:** 1 Department of Chemistry, Technical University of Denmark, Kgs. Lyngby, Denmark; 2 Department of Biomedicine, Aarhus University, Aarhus, Denmark; 3 Department of Molecular Medicine, Aarhus University Hospital, Aarhus, Denmark; 4 Section of Rheumatology, Medical Diagnostic Center, Silkeborg, Denmark

**Keywords:** rheumatoid arthritis, citrullinated peptides, tolerance recovery, anti-citrullinated protein antibodies, antigen-specific therapy

## Abstract

Rheumatoid arthritis (RA) involves a breakdown of immune tolerance to citrullinated proteins, leading to chronic inflammation and joint damage. Despite advances in treatment, achieving long-term remission remains a major challenge. Restoring immune tolerance to citrullinated proteins represents a promising strategy to halt disease progression and establish lasting remission. This review examines the potential of using citrullinated proteins or peptides to reestablish immune tolerance in RA. It explores the potential role of anti-citrullinated protein antibodies (ACPAs) in disease pathology and how utilizing or targeting specific citrullinated antigens could modulate immune responses. The review also highlights the therapeutic relevance of altering T and B cell function to regulate immune state. We explore mechanisms through which tolerance can be induced, including the use of citrullinated peptides to promote regulatory T (Treg) cell expansion and alter pathogenic B cell subsets. Emerging strategies aimed at re-educating the immune system are discussed, focusing on their potential to provide effective and durable treatment outcomes. These tolerance-based approaches are evaluated for their capacity to shift the immune response away from autoimmunity and towards sustained remission.

## 1 Introduction

RA is a chronic, systemic, autoimmune disease characterized by synovial inflammation and joint destruction, which affects approximately 18 million patients worldwide (1). Patients often grapple with fatigue, depression, and the fear of progressive disability, contributing to a lower quality of life (2, 3).

RA is subcategorized into seropositive and seronegative forms based on the serological presence of the autoantibodies rheumatoid factor (RF) and ACPAs (4–10). Approximately 70% of RA patients test positive for these antibodies (4–10). ACPA positivity is especially relevant, as this is associated with a more severe disease course and involvement of other organs (11, 12). ACPAs appear up to 10 years before disease onset (6, 12, 13) and they have great value in clinic practice as diagnostic tools (14).

Loss of immune tolerance is a central event in RA pathogenesis, leading to persistent activation of autoreactive T and B cells and production of autoantibodies such as ACPAs. Unlike conventional therapies that broadly suppress the immune system, restoring immune tolerance offers a targeted strategy to reprogram autoreactive responses while preserving protective immunity. Antigen-specific immunotherapies aim to achieve this by inducing T cell exhaustion, expanding Treg cells, or modulating antigen-presenting cells toward a tolerogenic profile. In recent years, several approaches -ranging from tolerogenic vaccines and peptide-based antigen presentation to therapeutic ACPAs- have shown promise in preclinical and early clinical settings. This review summarizes emerging strategies that leverage citrullinated peptides and other autoantigen-based interventions to restore immune tolerance in RA, with a focus on their mechanisms, efficacy and translational potential.

## 2 Breach of immune tolerance in rheumatoid arthritis

Rheumatoid arthritis is widely recognized as a T cell mediated disease. In genetically predisposed individuals, modified self-antigens can be presented via major histocompatibility complex (MHC) to self-reactive T cells, initiating a series of immunological events that progressively involves other cell types and ultimately leads to stablished RA. Preclinical stages are marked by expansions of distinct T cell subsets, including CCR2^+^ CD4^+^ T, T peripheral (Tph), T helper 1 (Th1) and CXCR5^+^ CD8^+^ T cells (15, 16). CD4^+^ T cells differentiate into multiple effector lineages -Th1, Th2, Th17 and T follicular helper (Tfh) cells- to coordinate immune responses. Imbalances in these subsets cause a proinflammatory response (17–20). Consistent with this, both early and stablished RA display elevated frequences of CD4^+^T cells in synovium compared to blood (15, 21, 22), accompanied to skewed Th cell profiles (15, 23). Th1 cells are involved in the release of proinflammatory cytokines (such IFN-γ, IL-2 or TNF-α), leading to bone erosion; while Th17 cells also stimulate the production of proinflammatory cytokines in synovial fibroblasts, with IL-17A being the predominant one. Similar alterations are observed in cytotoxic CD8^+^ T cells, with RA patients exhibiting different populations of CD27^−^CD62L^−^, CXCR5^+,^ GZMB^+^ and GZMK^+^ CD8^+^ T cell subsets, among others (15, 16, 24, 25).

While T cell dysregulation is central to RA pathogenesis, abnormal B cell subset composition and function are also closely linked to the breakdown of self-tolerance. Small subsets representing as little as 0.6% and 5% of blood B cells population as it is the case of B10 and B10pro cells, respectively, can have a major role in autoimmune regulation. Found within the CD24^hi^CD27^+^ B cell subpopulation, ex vivo B10 and B10pro cells were reported to negatively regulate monocyte related *in vitro* cytokine production through IL-10 dependent pathways (26). IL-10 knock out in B cells of collagen induced arthritis (CIA) murine models has also been shown to cause disease exacerbation characterized by an increase in inflammatory Th1 and Th17 cells, as well as a reduction in CD4^+^ T regulatory type 1 induced IL-10 production and increase in IL-17 levels (27). Other IL-10 knockout mice models such as the tamoxifen-induced model also proved the paper of IL-10 from Breg cells in CD4^+^ and CD8^+^ T cell mediated inflammatory cytokine expression (28). Subsequently, Aoun et al. reported natural autoreactive B cells specific for collagen type II C1 epitope (C1-B cells) present in the spleen, bone marrow and PBMCs of healthy mice, rats and humans, indicating its regulative role. However, RA patients showed an eight-fold decrease of C1-B cells while increasing the number of RA specific antibodies to C1 collagen epitope (29). Transfer of C1-B cells from anti-C1 mice into autoimmune prone mice model protected these against collagen type II arthritis induction (29). Antagonizing the previously described results on IL-10’s role in prevention of self-tolerance breach, IL-10 knockout C1-B cells from anti-C1 mice also suppressed collagen type II arthritis induction and increased activated T cells, pointing out that C1-B cells may tolerize T cells independently of IL-10 (29).

Autoimmune checkpoint molecule programmed cell death 1 (PD-1), expressed by T cells, B cells and other immune cells, plays a crucial role in maintaining immune tolerance and autoimmunity prevention by downregulating immune responses. Several publications pointed out the role of PD-1, its ligands or Cytotoxic T-lymphocyte Associated protein 4 (CTLA-4) overexpression, in T cell exhaustion (30–32), as well as synovium infiltration of PD-1^hi^Tph cells in early RA (21). Nettersheim et al. identified higher expression of PD-1 and CD73 in self-specific CD4^+^ T cells from healthy mice, compared to exogenous-specific CD4^+^ T cells (33). After blockade of both PD-1 and CD73, vaccine-expansion of self-specific CD4^+^ T cells resulted into CD4^+^ T cells with transcriptomes of exogenous-specific CD4^+^ T cells, showing that PD-1 and CD73 co-operationally limit CD4^+^ T to self-antigens (33). PD-1 and its ligands PD-L1 and PD-L2 expression has also been found upregulated in RA synovial tissue (34). Downregulation of PD-1 pathway was also observed during RA progression, attributed to increased levels of serum soluble (s)PD-1 in ACPA-positive (ACPA/+) RA patients (34, 35). sPD-1 was connected to severe CIA through Th1 and Th17 pathways (35), while PD-1 expression on CD4^+^ and CD8^+^ from PBMCs negatively correlated to disease activity (36). Further underlining the role of PD-1 in RA immune regulation, cases have been reported of RA occurring after PD-1 inhibiting cancer treatment (37). PD-1 can also drive T cells into apoptosis or a regulatory phenotype upon PD-L1, except in the case of RA patients (38). Generation of monocyte derived tolerogenic dendritic cells (tolDCs) with superior capacity to induce Th17 cells were obtained when precursor monocytes from peripheral blood of RA patients were treated with either P-selectin, IL-10 or PD-1 (39).

Upregulated levels of B cell activating factor (BAFF) in the peripheral blood was related to the survival of autoreactive B cells and further production of autoantibodies, exacerbating the disease (40, 41). Along with BAFF, toll-like receptor (TLR) ligands boost B cell activation, immunoglobulin isotype class switching, somatic hypermutation, and their transformation into plasma cells, which results in the production of harmful autoantibodies (42, 43). Likewise, *in vivo* studies on CIA mice indicate that silencing BAFF receptors expression lowers B cell counts and autoantibody levels significantly, which further reduces joint inflammation (44).

Furthermore, IL-6 produced by B cells and macrophages in the synovial fluid (SF) of RA patients, is needed for B cell differentiation and the formation of plasma cells (45). IL-21, secreted by subsets of helper T (Th) cells and found in higher levels on serum and SF of RA patients, is also essential for B cell activation, proliferation, differentiation and antibody production (46).

### 2.1 Environmental factors – smoking, neutrophil extracellular traps formation and role of mucosal immunity

**Figure 1:**
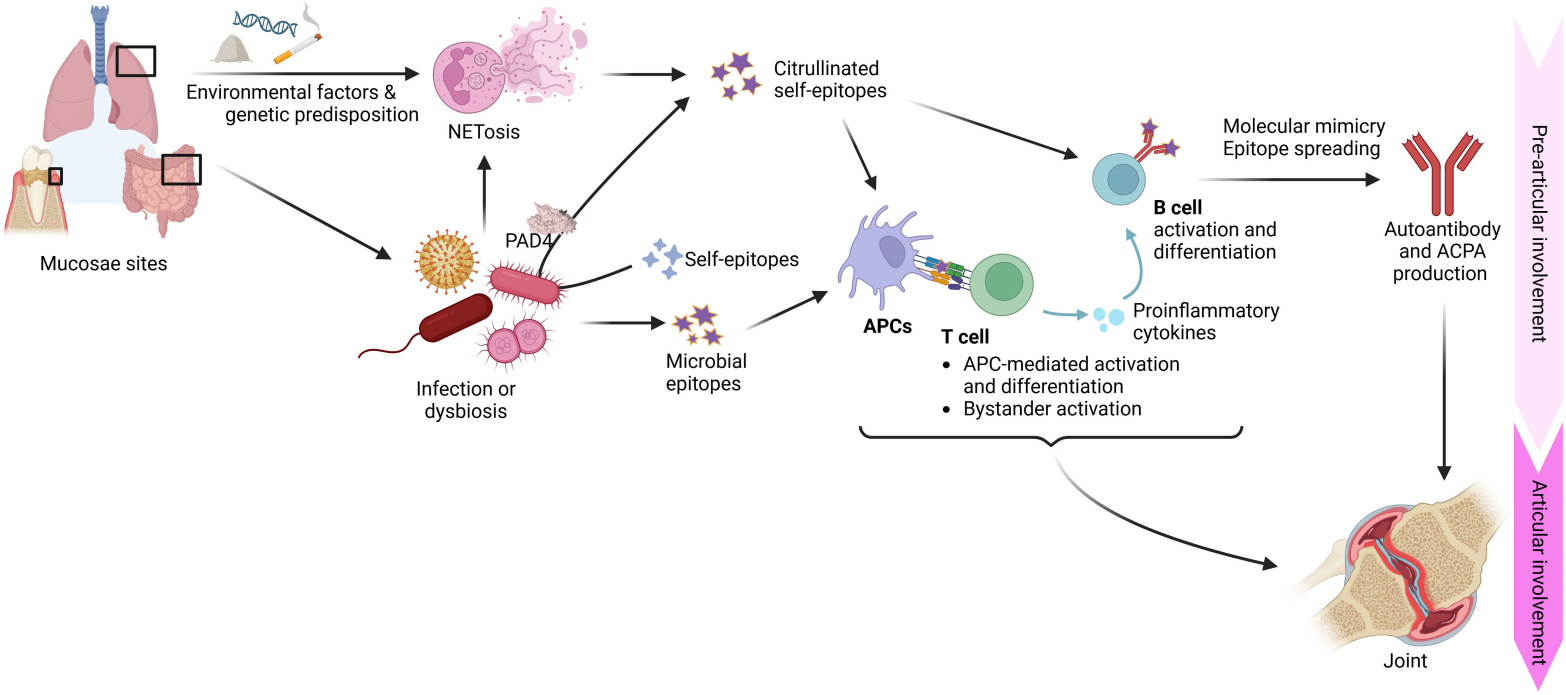
Representation of breach of tolerance during pre-articular stage in rheumatoid arthritis. Infective agents, environmental factors such cigarette smoking or some types of pollution and genetic factors mediate the loss of immune tolerance towards self-epitopes before disease onset. APCs, antigen presenting cells; NETs, Neutrophil Extracellular Traps; PAD, protein arginine deaminase. Created with BioRender.

The loss of immune tolerance in RA related to the impaired clearance and excessive presence neutrophil extracellular traps (NETs) has been previously reviewed (47–49). When NET removal is compromised, they accumulate at inflammatory sites, thereby prolonging inflammation and producing new autoantigens (47). Elevated NET formation has been observed in the sputum of both individuals at risk for developing RA (being first degree relatives of RA patients) and in RA patients themselves (50, 51). This local NET buildup correlates with the generation of mucosal autoantibodies such as IgA and IgG ACPAs, suggesting that the airway may serve as an initiation site for systemic autoimmunity. In fact, high levels of both NETs and ACPAs have been detected in the sputum of at-risk patients, supporting a direct association between NET formation and autoantibody production (50–53). Environmental factors such as cigarette smoking exacerbate this process by inducing NET formation via protein arginine deaminase (PAD) 4-dependent pathways, which in turn increases the production of citrullinated antigens in the lung (**Figure 1**) (54). Smoking not only elevates the risk of ACPA development but also intensifies the inflammatory response by triggering spontaneous NETosis in neutrophils (54–56).

In addition to environmental triggers, infectious agents have been noticed for their role in breaking immune tolerance (**Figure 1**) (57–59). Multiple pathogens -including Epstein-Barr virus (EBV), Mycobacterium tuberculosis (MTB), Porphyromonas gingivalis (Pg) and others- have been implicated as potential instigators of RA (57–59). Antibodies towards these infections and dysbiosis of mucosae’s microbiota have been found in higher titters on RA and early RA patients, compared to healthy controls (60–64). These microorganisms may trigger autoimmunity through mechanisms such as molecular mimicry, where the structural similarities between microbial antigens and self-proteins provoke a cross-reactive immune response to self-antigens; epitope spreading, which broadens the autoimmune response to additional self-antigens; and bystander activation, where infection-induced inflammation and cytokine release non-specifically activate T cells (65). Together with NET formation, these mechanisms expand the pool of autoreactive T and B cells, lowering the threshold for autoimmunity (48, 58). As an example, high sequence homology between these microorganism’s antigens and key host molecules like interferon regulatory factor 5 (IRF5), involved in macrophage and dendritic cells (DCs) inflammatory response as well as B cell antibody production, has been found (66). This similarity results in cross-reactivity towards antigens from EBV, MAP and self-IRF5 (66, 67).

The mucosal endotype hypothesis further explains RA pathogenesis by emphasizing the role of different mucosal sites: lungs, gut and oral cavity (58). Each of these sites exhibits unique inflammatory responses that may contribute to the systemic generation of autoantibodies. As it has already been covered, early inflammatory changes and local antibody production in lungs have been linked to both smoking and chronic respiratory infections. Similarly, in the oral cavity, periodontal disease driven by pathogens such as Pg not only damages the local mucosal barrier but also promotes NETosis and subsequent citrullination of bacterial as well as host proteins (68–70). This process can initiate a B cell response that eventually leads to the production of autoantibodies, setting the stage for joint inflammation (69). Moreover, the gut microbiome in patients with RA often shows distinct patterns of dysbiosis that are associated with metabolic changes and immune activation (71–73). These gut bacteria alterations can further contribute to the systemic inflammatory setting that underlies RA. Autoreactive B cells, generated autoantibodies or new self-epitopes resulting from these mucosal inflammatory processes can migrate systemically towards the joint (52, 74–79). Collectively, these observations underscore a multifaceted interplay between genetic predisposition and environmental attacks - including smoking, microbial infections and possibly even exposure to inorganic particles like silica (80, 81) - that collectively disrupts immune tolerance, culminating in the onset and progression of RA. Given the multitude of factors and mechanisms capable of breaking tolerance, RA is inherently a heterogeneous and complex disease.

Breach of tolerance is characterized by the aberrant presentation of citrullinated proteins, which primes both innate and adaptive immune responses, ultimately leading to the chronic production of inflammatory cytokines, autoantibodies and perpetuation of tissue damage. Consequently, restoring immune tolerance, particularly to citrullinated proteins, represents a promising therapeutic avenue for achieving remission in RA.

## 3 Role of ACPAs in pathogenesis of rheumatoid arthritis

While ACPAs are well established as diagnostic biomarkers for RA (14), increasing experimental and clinical evidence indicates that ACPAs are not just markers of disease but active contributors of joint pathology. Beyond their value in diagnostics, ACPAs are associated with disease severity or treatment outcome (82). In addition, epitope spreading reflects ongoing activation of autoreactive B and T cells, pointing towards their active role in disease (83).

The following section examines evidence on processes leading to the generation of citrullinated antigens that drive ACPA generation, the B-cell subsets involved in ACPA production, their structural diversity and the cellular and molecular pathways by which ACPAs contribute to synovial inflammation and joint destruction.

### 3.1 Generation of citrullinated antigens

Citrullination of proteins is a posttranslational modification consisting of the deamination of arginine by PAD enzymes. Under physiological conditions, this modification serves as a regulatory mechanism for protein function and is well tolerated by the immune system (84). However, when citrullination overcomes physiological regulation, changes in conformation and charge distribution of peptides leads to disrupted protein interactions, converting citrullinated epitopes into self-antigens (85).

Excessive citrullination can promote protein autophagy and subsequent presentation by DCs, macrophages and thymic DCs, driving CD4^+^ T cell activation (86). It can also enhance peptide binding affinity to MHC-II (87), leading as well to CD4^+^ T cell activation and contributing to tolerance breakdown.

Normally, dominant factors of self-antigens interact with the MHC-II of antigen presenting cells (APCs), while “cryptic” epitopes remain unrecognized. As a result, dominant epitopes become available for recognition during thymic T-cell tolerance, while a population of CD4^+^ T cells remain capable of recognizing cryptic epitopes. As it has been mentioned, citrullination can unmask these epitopes by increasing their MHC-II binding affinity, enabling their presentation and recognition by autoreactive T cells (85).

Interestingly, overcitrullination does not only disrupt tolerance when it happens on a self-antigen; PAD2 enzyme has been reported to citrullinate transcription factors responsible for CD4^+^ T cell differentiation into Th1, Th2 and Th17, altering the differentiation itself and the populations of the resulting type helper T cells (85, 88). Citrullination of cytokines CXCL10 and CXCL11 reduce their interaction with T cells, hindering their chemotaxis to inflammation site (85, 89).

Overall, there are subpopulations of both B and T cells reacting towards citrullinated epitopes in the synovial of RA patients, being the last ones commonly found mainly as Th1 and Th17 phenotypes (90–93). Several efforts have been carried out to identify pathogenic B cell subsets in ACPA/+ RA patients. By single cell RNA-sequencing of CD45^+^ hematopoietic cells, Wu et al. found differences between the synovial immune cell subsets of ACPA/+ and ACPA/- RA patients, pointing out different immunopathological mechanisms related to these autoantibodies (94). Aiming to find pathogenic B cell subsets, Thorarinsdottir et al. found that in ACPA/+ RA patients most of the B cells in SF belonged to a CD21^-/low^ subset. Under IL-6 stimulation, these cells expressed CXCR3 and RANKL, leading to osteoclast differentiation and bone destruction (95). Among this subset, ACPA/+ patients displayed CD21^-/low^CD27^-^IgG^-^ class significantly increased in peripheral blood and comprising 40% of the CD21^-/low^ cells in SF (95), matching posterior studies in which CD27^-^IgD^-^ and memory CD27^+^ IgD^-^ B cells were found in higher ratios in the SF compared to peripheral blood, suggesting these subgroups are key players in RA synovium inflammation (96). Consistent with previous results, Floudas et al. further proved the reduced presence of CD27^+^ IgD^+^ B cells along with the accumulation of PD-1^+^ B cells in SF and synovial tissue of RA patients, compared to healthy controls (97). Other subtypes found in higher percentages in ACPA/+ patients were CD19^+^ B cells (91); and for patients with early RA, human leukocyte antigen (HLA)-DR^+^-peripheral type helper T cells, PD-1^hi^ CD8^+^ T cells, CXCR5^−^ CD11c^−^ CD38^+^ naive B cells (98) and CD19^+^ CD24^hi^ CD38^hi^ regulatory B cells (99).

### 3.2 Diversity and glycosylation of ACPAs

ACPAs isolated from serum, plasma and SF of RA patients have been found as targeting over 100 citrullinated proteins (100–104). Notably, the affinities of these antibodies vary significantly both among patients and as disease progresses (83, 105–107). While some ACPAs only bind a single target such as citrullinated vimentin, fibrinogen or collagen (108, 109), most are highly promiscuous towards multiple citrullinated epitopes (110–112) or even other posttranslational modifications as acetylation and carbamylation (10, 112–114). Structurally, ACPAs are heavily N-glycosylated in their fragment antigen-binding (Fab) domain. Over 90% of ACPAs (compared to 15-25% of IgGs in human serum) are N-glycosylated in their variable domain (115, 116), and over 80% of receptors on ACPA-producing B cells contains N-glycosylation sites (116, 117). It is suggested that N-glycosylation provides ACPA-producing B cells with a selective advantage, enabling them to escape negative selection of the B cell receptor, thereby promoting autoimmunity (116, 118). On the other hand, Zhao et al. unveiled the complexity N-glycosylation in ACPAs by proving that upregulating sialylation of the crystallizable fragment (Fc) of ACPAs in B cells from collagen induced arthritis CIA mice attenuates disease progression (119), correlating to previous literature reporting decreased sialylation in the Fc region of serum ACPAs from RA patients and how this desialylation is related to inflammatory processes (119–122).

### 3.3 Mechanisms of ACPA-mediated pathogenesis

**Figure 2:**
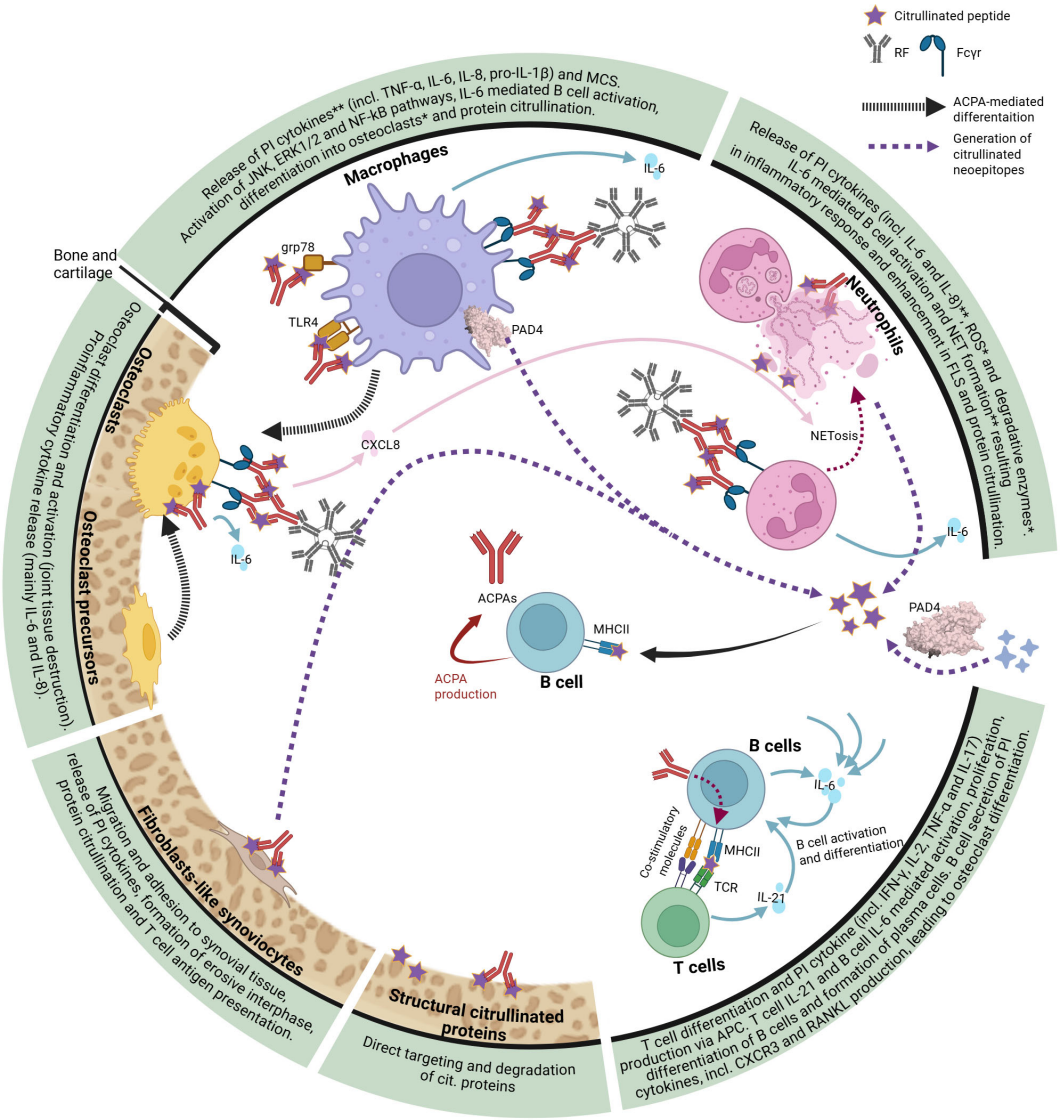
Representation of ACPA-mediated pathogenesis in synovium and crosstalk of different immune and structural components in seropositive RA. Main ACPA-derived response on different cell types is stated. APCs, antigen presenting cells; CXCR3, chemokine receptor 3; ERK, extracellular signal-regulated kinase; FcγR, Fc gamma receptor; FLS, fibroblast-like synoviocytes grp78, glucose regulated protein 78; JNK, Jun kinase; MHC, major histocompatibility complex; MCS, macrophage colony stimulator; NETs, neutrophil extracellular traps; NF-κB, nuclear factor-kappa B; PAD, protein arginine deaminase; PI, proinflammatory; RF, rheumatoid factor; ROS, reactive oxygen species; TLR4, Toll-like receptor 4; *reported via IC mediation; **reported both via IC and ACPA mediation. Created with BioRender.

The presence and pathogenesis of ACPA in murine arthritic models has been debated (108, 123). Their proposed pathogenic mechanisms include direct targeting and degradation of citrullinated proteins in joint cartilage, such as type II collagen; enhancing fibroblast-like synoviocyte migration and adhesion within the synovium, where they release proinflammatory cytokines, create an erosive interphase and are involved in the citrullination of new self-antigens (124, 125); direct targeting of osteoclast precursors promoting their differentiation (108, 126); or, as it will be further discussed, interaction with several immune system components resulting in a feedback loop that enhances the production of more ACPAs and proinflammatory agents such as cytokines, reactive oxygen species (ROS) and degradative enzymes, among others (**Figure 2**). Interestingly, ACPAs have different mechanisms when they interact on their own or via Fc gamma receptor (FcγR) after forming immune complex (IC) with RF (**Figure 2**). A protective role of ACPAs has also been suggested (127), highlighting the functional diversity of these autoantibodies.

Pointing out the effect of ACPA in different immune subsets, evidence shows that T follicular Th cells responses were reported higher in ACPA/+ than in ACPA/- (96). On the other hand, percentage of disease relevant Th17 was not dependent on seropositivity (91, 92).

ACPAs interaction with citrullinated glucose-regulated protein 78 (grp78) on macrophages’ surface reported activation of extracellular signal-regulated kinases (ERK)1/2 and c-Jun N-terminal kinase (JNK) signaling pathways, as well as enhancing NF-κB activity and tumor necrosis factor-alpha (TNF-α) secretion (128–130). ACPAs can also activate macrophages via TLR4- and MyD88- dependent (131–133) or CD147-integrinb1-PI3K-Akt pathways, this last one activating NF-κB signaling and NLRP3 inflammasome cascade and pro-IL-1β release (134). Otherwise, when found as ICs with RF, monocytes were also stimulated by binding FcγRs which enhanced proinflammatory cytokine release in synovial membrane (135) as well as regulating differentiation into osteoclasts (136, 137). Breedveld et al. stimulated monocytes with SF isolated ICs, resulting in IL-6 and IL-8 release and subsequent activation of osteoclast activation (133, 138). Connection of PAD4 and macrophages in RA has been described. Enzymatically active PAD4 was found present on the monocyte surface, being a source of novel ACPA autoantigens by citrullinating both soluble and surface proteins (139). These findings correlate with the already reported role of SF and lymphoid tissue macrophages in citrullination of proteins and ACPA production (140). Interestingly, autocitrullination of PAD4, which is found in SF ACPA/- patients, exacerbated inflammatory arthritis in mice models through monocyte recruitment, suggesting an ACPA-independent role of PAD4 in RA pathogenesis (141).

Neutrophiles are another immune cell type targeted by ACPAs (142, 143). The already mentioned ICs have been reported to activate neutrophiles leading to cartilage and tissue destruction due to neutrophil degranulation, release of degradative enzymes, ROS, as well as activation of soluble receptors and cytokines causing general tissue inflammation (144, 145). NET formation has also been observed on SF and sera of RA patients (146–149), correlating to ACPA levels and their immune complexes, which enhances inflammatory response in synovial fibroblasts via activation of IL-6, IL-8 and adhesion molecules, among others (138, 147–149). Some forms of NETosis rely on PAD4 activity (150, 151) and results into citrullination of proteins (specially histones) in the synovial space, engaging a positive feedback loop for which either synovial autoreactive ACPA-producing B cells or direct presentation of citrullinated antigens to T cells by fibroblast-like synoviocytes leads to the production of more autoantibodies (148). Indirectly, neutrophils can also get activated through ACPA binding to osteoclasts, as this leads to secretion of CXCL8, promoting neutrophil attraction and NET release, which again increases ACPA activity through binding to citrullinated histones in the released NETs (152).

Even though the appearance of ACPAs has been linked to environmental factors as smoking and some viral infections (among others) (153, 154), ACPA/+ RA patients have shown a gene signature based on the already mentioned HLA complex, which is crucial for antigen presentation between immune cells. Both RA and ACPA development were found to be connected to HLA haplotypes expressing the shared epitope (SE), which codes for a QKRAA peptide motif on the MHC (153–157). Similarly, both humanized and non-humanized mice models expressing different RA-related haplotypes of HLA containing the SE generated ACPAs to a greater extent upon disease induction with PAD rather than those with haplotypes lacking SE (158, 159).

## 4 Current treatment landscape for rheumatoid arthritis

Current pharmacologic treatment options for RA can be divided into three major groups: steroids and disease-modifying antirheumatic drugs (DMARDs). Steroids are only symptomatic and are not able to change the long-term course of the disease, therefore the European League Against Rheumatology (EULAR) recommendations for RA treatment utilizes conventional synthetic DMARDs (csDMARDs) methotrexate (MTX) as initial treatment, eventually in combination with short-term glucocorticoids during disease flares. In the case that csDMARDs are not effective, biological DMARDs (bDMARDs) (which are related mainly to cytokine regulation) or targeted synthetic DMARDs (tsDMARDs) such as Janus Kinase (JAK) inhibitors are employed. Here, multiple modes of action are available, underlining the immunopathological heterogeneity of RA (160). As it is illustrated in different cohorts, most of RA patients receive MTX while smaller fraction receive bDMARDs (161–163).

Treatments are aimed to reduce disease activity and prevent joint damage; managing RA follows treat-to-target strategies (164). If treatment target (which is based on remission in early disease and low disease activity in long-standing disease), is not achieved at 3 and 6 months, respectively, EULAR guidelines (2022) recommend a change in treatment regime.

The persistence of different groups of therapeutics in clinic has been reviewed over the last 6 years by several nation-wide organizations, with cohort ranging from 900 to 5100 RA patients. Depending on the cohort, TNF-α inhibitors (bDMARDs targeting TNF-α) have a retention rate of 29% to 58%, while in the case of JAK inhibitors, retention rates are 40% to 72% (165–167). Still, discontinuation rates due to adverse events are similar between TNF-α inhibitors and JAK inhibitors (168).

### 4.1 Challenges in achieving immunological remission

Existing treatments such as MTX and several costly biological therapies can slow disease progression but do not cure the disease. Depending on the cohort, range from 39% to 70% patients do not reach the preferred goal of sustained remission or low disease activity (161–163).

The effect of bDMARDs and JAK inhibitors can also be dependent on ACPA seropositivity. For example, drugs like the JAK inhibitor tofacitinib (169), B cell depletor rituximab (170, 171) and T cell modulator abatacept (172) have better efficacy on seropositive groups, compared to seronegative groups. TNF inhibitors show similar efficacy in seropositive and seronegative disease (172).

In a cross-sectional analysis of RA patients treated with various csDMARDs and/or JAK inhibitors, Neppelenbroek et al. suggested that ACPA^+^ B cells retained their activated and proliferative phenotype, despite effective control of inflammation and clinical disease. The absence of immunological remission might explain why ACPA/+ patients rarely reach sustained drug-free remission. This continued activated state of ACPA-B cells indicates chronic exposure of these cells to stimulating triggers along disease course, which in this study was 11 years (average) (173). Tocilizumab, another FDA-approved bDMARD, managed to decrease synovial T cells and disease activity on patients after 8 weeks of treatment, but did not manage neither to decrease the count of CD68^+^ macrophages or CD20^+^ B cells in synovium, maintaining unchanged local levels of RANKL and significantly increasing systemic levels of IL-6 and RANKL (174), two cytokines that as previously mentioned, are expressed by synovial macrophages and B cells and are related to joint erosion (45, 133, 138).

Despite significant advances, current RA therapies do not achieve durable, immunological remission across all patient groups. Their effectiveness often depends on ACPA status, with seropositive individuals responding more favorably, yet still without showing immunological remission despite clinical improvement. To address this disparity, emerging antigen-specific therapeutic strategies are proposed as considerable therapeutics toward sustained immunological and disease-modifying remission.

## 5 Restoring immune tolerance: emerging mechanisms and therapeutic approaches

### 5.1 Fundamental mechanisms of tolerance restoration

Tolerogenesis or tolerance recovery is understood as the process by which the immune system re-establishes its ability to recognize and tolerate self-antigens, thereby preventing autoimmune responses and maintaining immune homeostasis. Mechanisms of immune tolerance can be broadly divided into central and peripheral tolerance. Central tolerance occurs primarily in the bone marrow and thymus, where autoreactive T cells undergo clonal deletion before entering the circulation (175, 176). Peripheral tolerance, by contrast, regulates mature T cells in the periphery through multiple mechanisms, including (i) T cell anergy, where T cells become non-proliferative upon antigen stimuli, commonly lack co-stimulatory molecules and are functionally inactive (177, 178); (ii) T cell ignorance, being ignorant T cells unresponsive to their autoantigens yet potentially able to be activated again (179); (iii) T cell exhaustion, associated with constant antigen exposure (180, 181); (iv) clonal deletion of mature T cells in the periphery, mediated through antigen presentation (182, 183).

Treg cells can suppress local immune responses elicited by Th cells upon receptor activation of disease-causing antigen (184, 185). Additionally, Treg expansion has been proved to reinduce tolerance (177, 186, 187). Highlighting the pivotal role of antigen-specific Treg expansion in tolerance recovery, imbalances in Th1/Treg and Th17/Treg (as well as Th1/Th2 ratios) are often associated with loss of tolerance in RA (17–20).

### 5.2 Established therapies with tolerogenic potential

**Figure 3:**
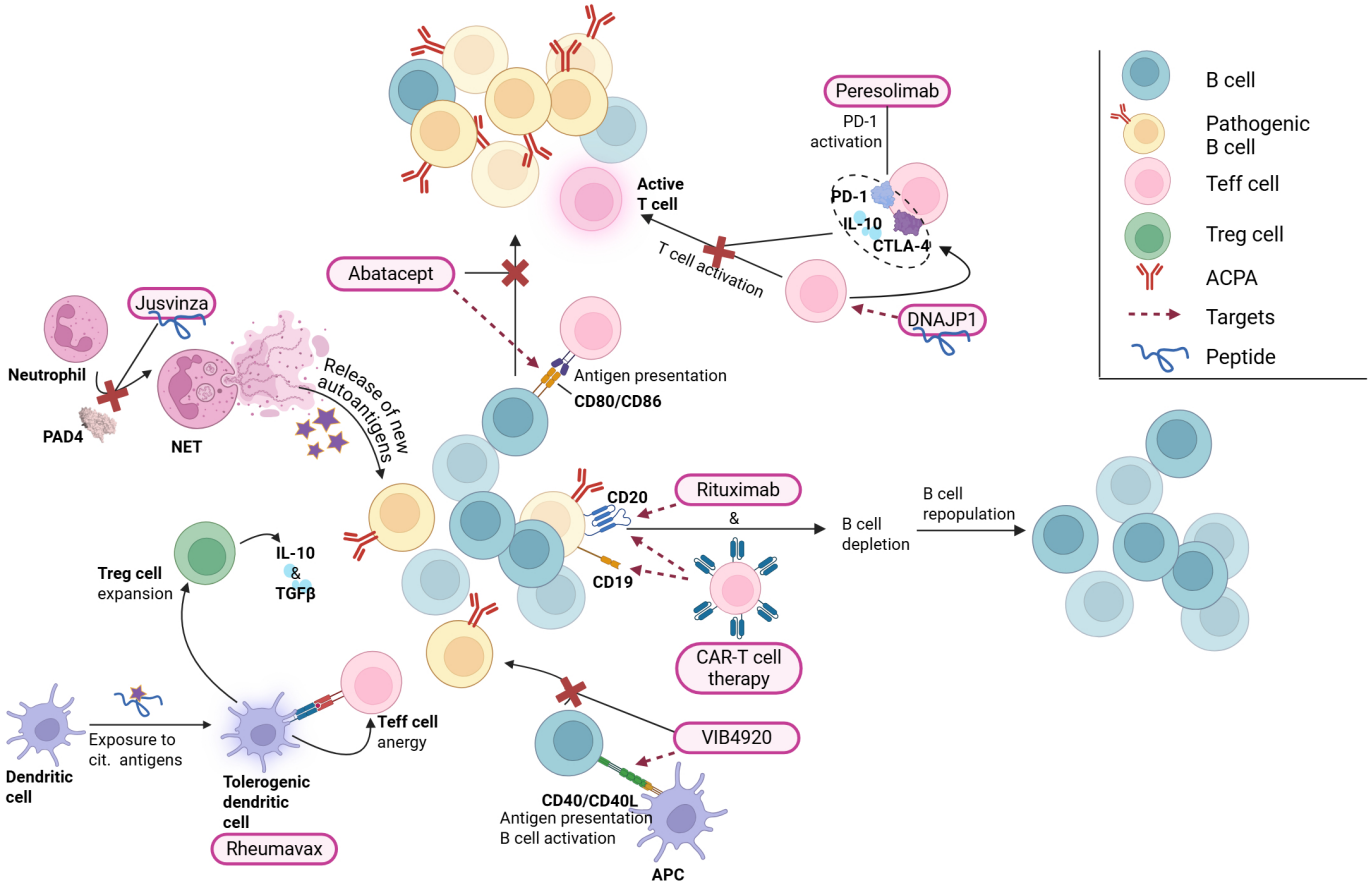
Principal mode of action of some of the tolerogenic drugs and drug candidates reviewed in this work. APCs, antigen presenting cells; CAR, Chimeric Antigen Receptor; CTLA-4, Cytotoxic T-lymphocyte Associated protein 4; PD-1, Programmed death cell protein; NETs, Neutrophil Extracellular Traps. Created with BioRender.

The bDMARD abatacept targets CD80 and CD86 on the surface APCs including B cells. CD80 and CD86 are key co-stimulatory molecules for antigen presentation and T cell activation. In a study by Lorenzetti et al, *in vitro* abatacept treatment was shown to decrease CD80-CD86 expression on B cells in a dose-dependent manner. In contrast, clinical assessment revealed only a moderate reduction in ACPA levels but a significant decrease in the ACPA-specific B cell population, suggesting a restoration of tolerance (**Figure 3**) (188). The bDMARD rituximab targets CD20, leading to a depletion of B cell populations for 6-9 months (189), yet without elevating the likelihood of infections in patients relative to other forms of bDMARD treatment (190, 191). Tolerance recovery is suggested by the posterior regeneration of the B cell subpopulations, finding different subset composition than found before treatment (**Figure 3**) (192). Naïve B cell population increased, while CD27^+^ memory cells stayed significantly reduced (0.5-fold) for up to 2 years (192).

## 6 Novel drug candidates and strategies for tolerance induction

Current RA treatments do not effectively target the underlaying immunologic causes of the disease, being reflected in the high relapse incidence and the large RA population that does not achieve complete remission. One study found that after 5 years of treatment, 55% of RA patients had switched treatment due to treatment failure or, to a lesser extent, due to adverse events (193). Given RA heterogeneity and that patients may require multiple successive therapies throughout life (160), there is a need for treatments employing different modes of action. This need is reflected in the several trials for RA treatment that have been reported during the last 10 years, where the main goal is to restore tolerance and “re-educate” the immune system rather than decrease inflammation by targeting its components (**Figure 3**, **Table 1**).

**Table 1:** Overview of therapeutic candidates discussed in this review with tolerogenic effects, either approved or under clinical investigation for RA.

Drug candidate	Type of therapeutic	Regulatory status	Mechanism/target	Formulation and dosage	Cohort size [ACPA/+ patients]	Outcome		Ref
						Inflammation reduction	Immunomodulatory regulation	
Abatacept	bDMARD	FDA- and EMA- approved	Inhibition of antigen presentation and T cell activation via CD80/CD86 expression downregulation.	Infusion, 10 mg/kg	9 RA refractory patients (DRKS00012864) (78% under prednisone, 100% under MTX) [100% ACPA/+]	Reduction in DAS28 score to low disease activity or remission ranges (< 3.2 and < 2.6).	Leucocytes and lymphocytes did not significantly change. Significant decrease (1.55-fold) in ACPA-specific B cell populations sustained over observation time (42 months).	(188)
Rituximab	bDMARD	FDA- and EMA- approved	B cell depletion via CD20 targeting.	Infusion 4x375 mg/m^2^ (weekly) or 2x1000 mg (every two weeks)	17 RA refractory patients (70% patients were under MTX)	DAS28 score improved from 6.1 to 4.1; CRP levels were reduced 80% from baseline.	B cell depletion for 6-9 months and posterior regeneration of naïve B cells. CD27^+^ memory cell population remained reduced (<50%) after regeneration for more than 2 years.	(192)
CAR-T cell therapy		[-]	CD-19 targeting and secretion of antibodies against IL-6 and TNF-α.	Cell infusion, 1.0×10^6^ CAR T-cells/kg	3 RA patients, (under csDMARD and bDMARD treatments) [100% ACPA/+]	Number of swollen joints decreased for all patients. DAS28-CPR scores decreased up to 66%, 48% and 34% of the initial value. No relapse was observed during observation period-9 months.	CD19^+^ B cell depletion and reduced IL-6, TNF- α, RF (not found after treatment) and ACPAs (35,6- to 865-fold) levels. RA-related antibodies were significantly reduced for 6 months. B cell reconstitution was observed from 60 to 90 days.	(194)
Rheumavax	Ex vivo-generated autologous tolDCs	Phase I (ACTRN12610000373077)	Reduction in T_eff_, potentially via clonal deletion or anergy.	Intradermal	34 RA patients: - 9, 1x10^6^ DCs - 9, 5x10^6^ DCs - 16, placebo (under TNFi and/or csDMARDs) [100% ACPA/+]	Treated patients with active disease (DAS28 > 2.4), DAS28 had a median change of -0.84 in 1 month and -0.45 in 6 months.	Reduction in Teff population by at least 25% in 11 out of 15 treated patients. Increase in Treg population by ≥25% in only 5 of 15 treated patients. Increase in Treg/Teff ratio by ≥25% in 11 of 15 treated patients.	(195)
Dazodalibep	Binding protein	Phase II, complete (NCT04163991)	B cell activation inhibition via CD40 targeting.	Intravenous infusion	57 RA patients: - 12, 1500mg - 12, 1000 mg - 10, 500 mg - 8, 75 mg - 15, placebo [96% ACPA/+]	DAS28-CRP score improved with a 2.3-2.2 point reduction for groups treated with the highest doses over 12 weeks.	Reduction in B cell proliferation (specially within IgD^−^CD27^+^ memory B cells) and T cell dependent antibody production upon immunization with keyhole limpet hemocyanin (KLH) two weeks after treatment. 43 days after immunization, anti-KHL IgG titters were undetectable for 15000 mg dosed group.	(196)
					78 RA patients: - 15, 2x3000mg - 16, 1x3000mg - 15, 4x1500mg - 16, 2x1500mg - 16, placebo	DAS28-CRP score showed improvement (1.83 to 1.90 points) in all treatment groups at day 113; reductions from DAS28-CPR baseline were also present at day 309.	[-]	(197)
IRL201805	Antibody	Phase II (RVLO 221-02)	Antigen presentation capacities of DCs reduction via inhibition of HLA-DR and CD86 expression.	Intravenous infusion	24 RA refractive patients -6, 15 mg -6, 5 mg -6, 1 mg -6, placebo (under csDMARDs)	DAS28 score reduction bellow 3.2 after 12 weeks in responding RA patients (43%).	Overexpression of CD39 in Tregs (related to Treg activity) in responding patients, after 12 weeks.	(198, 199)
Jusvinza	Peptide	Phase III (RPCEC00000433, RPCEC00000404)	PAD-4 mediated inhibition of NETosis.	Subcutaneous 4 doses/weekly, 5 doses/monthly	20 RA patients -6, 1 mg -5, 2.5 mg -9, 5 mg [60% ACPA/+]	DAS28 score reduction greater than 1.2 for all patients by end of observation period (6 months); as well as achievement of ACR50 and ACR70 by 28% and 61% of patients, respectively.	Significant reduction in serum ACPA levels. Ex vivo assays showed a 1.5-fold increase of Treg cells when PBMCs of RA patients were exposed to Jusvinza.	(200–202)
DNAJP1		Phase II, (NCT07013110)	Antigen specific tolerance recovery to HSP epitope and shared epitope homologue.	Oral, 6 months/daily.	160 active RA patients with immunologic reactivity towards DNAJP1 -36, 25 mg -45, 25 mg + HCQ -33, placebo -46, placebo + HCQ	At approximately 6 months, 33% and 49% of patients treated with DNAJP1 and HCQ achieved ACR50 and ACR20, respectively.	Alterations in T-cell differentiation clusters associated with self-reactivity, decreased TNF-α and increased IL-10, PD-1 and CTLA-4 expression.	(203)
DEN-181		Phase I, complete (ACTRN12617001482358)	Antigen specific tolerance recovery.	Liposomal injection containing CII-derived peptide and NF-κB inhibitor calcitriol, subcutaneous	56 RA patients under MTX treatment. - 4, 12.6 mg CII - 3, 42 mg CII - 4, 126 mg CII - 6, placebo [100% ACPA/+]	Patients on low and medium dose had a remission of < 2.6 DAS28-CRP on day 57. DAS28-CRP for patients treated with high dose increased over the first 15 days.	Reduction on citrullinated vimentin-reactive T cells and memory B cell, along with serum ACPA levels. Increase of CII-specific PD-1^+^ T cells within 28 days of treatment and identification of T cell transcripts relating to TCR signaling and T cell exhaustion.	(204)

CII, collagen type II.

### 6.1 Cell-based tolerance recovery therapies

In a small trial consisting of three patients with treatment resistant RA, CD19-directed Chimeric antigen receptor (CAR)-T cell treatment caused B cell depletion and reduced the pathogenic interleukins IL-6 and TNF-α as well as RF and ACPA levels (194). Along with lowering joint inflammation and the absence of relapse, the progressive repopulation of B cells non-associated to an increase of pathogenic antibodies 9 months after treatment makes CAR-T therapy a promising tool to restore tolerance in difficult to treat cases (**Table 1** for detailed information) (194). A reported case with this outcome described one RA patient treated with CD20/CD19-directed CAR-T cell therapy following a diagnosis of diffuse large B cell lymphoma (205). However, most available data for this type of treatment in RA come from very small cohorts, sometimes down to individual case reports (206, 207). *In vitro* data of similar CAR-T cell therapies backs up the previous results by eliminating autoreactive B cell populations from RA patients’ serum (208). However, longer and bigger trials need to be carried out to dismiss the serious toxic effects that this type of therapy is suspected to have in rheumatic autoimmune disease treatment (206, 209).

Ex vivo-generated autologous tolDCs introduced to a specific antigen have been explored due to their capacity to present antigens to T cells (210). TolDCs are not only able to cause T cell anergy or the expansion of Treg cells by providing constant exposition to the specific antigen in CIA murine model (211, 212), but they also express PD-1 and anti-inflammatory cytokines IL-10 and IL-35 (213). Thus, it is understandable that peptide loaded tolDCs have been successfully utilized in multiple clinical trials aimed at restoring tolerance in autoimmune diseases such as multiple sclerosis (214) and type I diabetes (215, 216). In the case of RA treatment, it is important to mention the few candidates that showed promising results in small cohort clinical trials phase I (9 to 18 patients), currently recruiting for further studies or ongoing clinical trials: Rheumavax (ACTRN12610000373077), AuToDeCRA (ISRCTN14999554) and CreaVax-RA (KCT0000894). AutoDeCRA tolDCs have been exposed to the antigens of the patients’ SF, while CreaVax-RA tolDCs have been exposed to PAD4, RA33 (heterogeneous nuclear ribonucleoprotein A2/B1 (hnRNP A2/B1)), citrullinated filaggrin and vimentin (217). AutoDeCRA trial failed to show efficacy on clinical inflammation parameters, changes in serum cytokine levels or in peripheral T cell phenotype (218). In the case of Rheumavax, consisting on tolDCs with the NF-κB pathway inhibited and exposed to citrullinated peptides derived from vimentin, collagen type II, aggrecan and fibrinogen, better results were observed. 1 month after Rheumavax treatment, T effector (Teff) cells were reduced compared to untreated controls, while the ratio of Treg/Teff increased, pointing to a shift in the immune balance (195). Cytokine IL-15, IL-29, CX3CL1 and CXCL11 levels as well as T cell mediated IL-6 response towards the citrullinated vimentin peptide found in Rheumavax were reduced (195). It is also worth mentioning the TOLERANT clinical study, which is in recruiting stage (phase I, NCT05251870). As well as the previously mentioned therapies, in this trial HSP70 peptide loaded DCs will be employed in order to induce and/or expand Treg populations (219).

### 6.2 Tolerogenic monoclonal antibodies and binding proteins

Peresolimab, an IgG monoclonal antibody that stimulates PD-1 pathway, showed a positive primary outcome by reducing DAS28 score compared to the placebo group 12 weeks after treatment in a phase II clinical trial. However, in secondary outcome measures, peresolimab was only significantly better than placebo with respect to ACR20 responses, but not with respect to ACR50 or ACR70 responses (220). Aiming for T cell activation suppression, peresolimab is intended to reset the immune response to restore immune tolerance (221). Differently, inhibiting B cell activation and plasma cell differentiation by means of CD40L binding protein targeting, dazodalibep was tested in a phase I trial (196). CD40 is expressed on many APCs (incl. DCs, macrophages and B cells) and non-hematopoietic cells. Effective humoral response to T cell-depending antigens rely heavily on CD40/CD40L interactions between B cells and T cells. Not only DAS28-CRP went down to -2.3 compared to baseline, but a significant reduction in B cell proliferation and T cell-dependent antibody production were reported (196). Further clinical trial (phase II, NCT04163991) confirmed the reduction of DAS28-CRP score over 309 days on a bigger cohort of 62 treated RA patients and 16 disease controls (197).

Part of HSP70 family, binding immunoglobulin protein (BiP) is involved in the peripheral blood monocytes differentiation into DCs and osteoclasts. Treatment of maturing monocytes with BiP results in reduced antigen presentation capacities of DCs due to lower expression of HLA-DR and CD86. Recombinant human BiP administration has been reported to prevent and ameliorate disease in murine CIA models (222, 223). BiP analogue IRL201805 was administrated to RA patients and its effects were monitored for 12 weeks in a phase I/IIa trial. DAS28 score was consistently reduced in the fraction of patients that responded to treatment (43%) (198) without any serious adverse drug reactions reported (199). When the 4 week-after treatment PBMCs of responder RA fraction was incubated with their own PBMCs before treatment, these ones produced significantly less IFN-γ than RA patients treated with placebo. As a part of the inflammatory response regulation, serum levels of pro-inflammatory cytokines IL-1β, TNF-α and IFN-γ were reduced while sCTLA-4 increased. Related to the pro- to anti-inflammatory shift observed in serum cytokine levels, Treg stability and potency related CD39 was found overexpressed in the Tregs of the patients responding to the treatment (198).

### 6.3 Peptide and antigen-based immunomodulatory therapies: emphasis on citrullination

Peptides emerge as highly specific and versatile drug candidates for tolerance recovery in RA and other autoimmune diseases (224–231). Their high specificity minimizes potential drug–drug interactions, making them suitable for combination with other RA therapeutics. Preliminary and exploratory trials in RA patients have employed them in combination with different csDMARDs reporting treatment efficacy and no concerning adverse effects (200, 203, 204). Compared to antibodies, their small size, enhanced stability, scalable production and customizable structure make peptides particularly attractive for achieving precise interactions with immune targets while maintaining relatively low immunogenicity and lower production cost characteristic of small molecules (232, 233).

The T cell-activating peptide based on the immunogenic HSP60 Jusvinza, approved in Cuba for cases of COVID-19 with hyperinflammation, is currently under clinical trials (phase III, RPCEC00000433) to treat RA patients. As it has been mentioned previously, NETosis is related to inflammation in RA as well as citrullination of new antigens and production of new ACPAs. Protein expression of neutrophiles from patients treated with Jusvinza was found to be differently modulated, including differences on the already mentioned NF-κB pathway (234). Overall, RA patients treated with Jusvinza displayed PAD4-mediated inhibition of NETosis, which was further confirmed with *in vitro* experiments (234). Phase I clinical trials in RA patients treated with Jusvinza showed a reduction on blood ACPA levels DAS28 score and achievement of ACR50 and ACR70 in 6 months (200, 201). Complementary, ex vivo assays showed a 1.5-fold increase of Treg cells when PBMCs of RA patients were exposed to Jusvinza (202), suggesting that tolerance recovery towards citrullinated antigens could be mediated by NETosis inhibition. Also derived from an HSP, T cell proinflammatory epitope DNAJP1 peptide was used to treat RA patients with PBMCs reactive towards the candidate (75% of the tested participants) in a clinical trial phase II (203). Upon treatment, TNF-α expressed by T cells decreased significantly while IL-10 expressed by T cell increased, along with PD-1 and CTLA-4 (203). These results match previous phase I outcomes for the same peptide, where DNAJP1-specific T cell number did not change over treatment (hence, there was no clonal deletion) but changes on clusters of differentiation on them pointed that immune reactivity towards the self-peptide did (235).

DEN-181, a subcutaneous formulation consisting of RA-joint HLA-DRB1*04:01- and *01:01-haplotypes specific collagen type II_259-273_ peptide and NF-κB pathway inhibitor calcitriol in liposome formulation, reduced the population of citrullinated vimentin-specific T cells in MTX-treated patients under a phase I clinical trial (204). The improvement in disease activity observed in RA patients was associated with the tolerogenic effects of the peptide-based therapy DEN-181, including an early expansion of PD1^+^ collagen type II– and citrullinated vimentin–specific T cells, followed by a reduction in ACPAs, an increase in CCR7^+^ naïve T cells and a decrease in memory B cells (204). CCR7^+^ expression in T cells is related to T cell migration from peripheral tissue to lymph node (236). Disruptions in this migratory process lead to peripheral tissue Teff cell accumulation in inflammation and autoimmunity, incl. RA (237). Used in DEN-181, calcitriol is a metabolite of vitamin D. Vitamin D has been reported to elevate the percentage of Tregs and lower the DAS-28 score just after 3 months of supplementation along MTX and hydroxychloroquine in RA patients, compared to a group that were just treated with the csDMARDs (238).

Citrullinated antigens have also demonstrated potential in promoting tolerance recovery. This process is typically achieved through the persistent exposure to the antigen via repeated administration of low doses, aiming to modulate T cell population by depleting or causing T cell exhaustion on pathologic Th1 and Th17 cells, reducing the expression of proinflammatory cytokines that mediate these, or increasing the population of Tregs (239). Gertel et al. utilized a multiepitope citrullinated peptide, containing motifs from key citrullinated proteins in RA such as filaggrin, fibrinogen, vimentin and collagen. Their approach successfully improved the clinical status of adjuvant-induced RA rats (**Table 2**). The increase of Treg and reduction in Th17 cells, previously associated with reactivity towards citrullinated epitopes (90–93), indicated tolerance induction (239).

**Table 2:** Summary of preclinical studies evaluating tolerogenic peptides and monoclonal ACPAs in murine RA models.

Therapeutic agent	Animal model	Study duration	Outcome		Ref
			Inflammation reduction	Immune cell and related cytokine regulation	
Multiepitope (*filaggrin, fibrinogen, vimentin and collagen*) citrullinated peptide	AIA rat	29 days	Mean paw diameter of treated group was 19% (*p* < 0.002) and 11% (*p* < 0.03) smaller than untreated and non-citrullinated multiepitope peptide controls, respectively. Trend was confirmed over inflammation indices measured with H&E immunostaining.	Significant increase in splenic CD4^+^ CD25^+^ Foxp3^+^ Treg populations was observed in treated rats, compared to untreated and non-citrullinated multiepitope peptide controls (*p* < 0.01). Parallelly, splenic IL-17^+^ CD4^+^ T cells (Th17) were significantly reduced in treated group compared to untreated controls (*p* < 0.03).	(239)
CEL-4000 (*proteoglycan epitope + CD4^+^ T cell ligand*)	GIA mice	35 days	By the end of observation period, arthritis score* (based on swelling and redness of paws, visual) for CEL-4000 and CEL-5000 treatment groups was lower than in adjuvant treatment control group (p = 0.0114 and 0.0671, respectively).	Cytokine profile production switch from Th1 and Th17 to Th2 and Treg. Compared to adjuvant treatment control, splenic cells from CEL-4000 mice had elevated IL-10:IFN-γ ratios (p ≤ 0.05); while in CEL-5000, elevations on IL-4:IFN-γ and IL-4:IFN-γ ratios were observed (p ≤ 0.05).	(240)
CEL-5000 *(citrullinated proteoglycan epitope + CD4^+^ T cell ligand)*					
CPEP2 *(Cyclic citrullinated fibrinogen derived epitope formulated in chitosan-based nanoparticle)*	CIA rat	56 days	At study termination, arthritis score* of rats treated with CPEP2 was 90% lower than control groups treated with PBS, MTX or unloaded nanoparticles.	Upon treatment with CPEP2, TFN-α serum levels were decreased compared to disease control. Serum and synovial fluid levels of IL-10 were found increased as well.	(241)
Citrullinated peptides from human cartilage intermediate layer protein (CILP) or fibrinogen	HLA-DR mice	14 days	[-]	Expansion of CD4^+^T cells binding to HLA-DR:citrullinated peptides; decrease of Th1 cells and increase of Treg cell populations.	(242)
ACPAs isolated from RA patients	CAIA mice	14 days	Compared to controls, different recombinant ACPA administration 3 and 7 days after CAIA induction reduced both paw thickness and disease severity (1.4 fold, p ≤ 0.001) and bone erosion, synovitis, and cartilage damage (p ≤ 0.01) regardless of ACPA specificities; preventing overall break of tolerance.	[-]	(127)
		28 days	Monoclonal ACPAs derived from patients did not show arthritogenicity nor pain signals on mice. ACPA clones E4 reduced osteoclastogenesis and protected mice from CAIA induction using different arthritogenic cocktails.	Clone E4 strongly binds to macrophages and RA proteins from synovial fluid as α-enolase, resulting in increased IL-10 secretion by macrophages (p = 0.0013).	(243)
		14 days	Several monoclonal ACPA inhibited CAIA or quantitatively ameliorated disease 2 days post-injection, if administrated at disease peak.	[-]	(244)
		13 days	When NETosis inhibitor monoclonal ACPA was injected 3 days posterior of arthritogenic antibody cocktail, arthritis score was reduced up to 94% compared to disease control (*p* < 0.01).	[-]	(245)

*based on swelling and redness of paws, visual; AIA, Adjuvant-Induced Arthritis; GIA, glucose-6-phosphate isomerase – Induced Arthritis; CIA, Collagen-Induced Arthritis; CAIA, Collagen Antibody-Induced Arthritis.

CEL-4000 consists of a proteoglycan (PG) non-citrullinated epitope derived from cartilage PG aggrecan (PG70) conjugated to a ligand specific for CD4^+^ T cells. This design allows the T cell presentation of the immunomodulatory peptide to an APC via MHC II while the CD4^+^ ligand modulates T- cell activity. CEL-4000 was tested in PG-induced arthritis (PGIA) and G1 domain-induced arthritis (GIA) mice models, switching cytokine production from Th1 and Th17 pro-inflammatory (TNF-α, IL-17 and IFN-γ) signature to an Th2 and Treg anti-inflammatory (IL-10, IL-4 and TGF-β) one, as well as an increase in Treg cells (240, 246). CEL-5000, which introduces a citrullinated PG epitope was also tested in PGIA and GIA mice models. CEL-4000 and CEL-5000 developed different immune responses, since mice did not produce high antibody titters for the citrullinated epitope conjugate while they did for CEL-4000’s. However, both treatments lowered arthritic score, reduced inflammation levels (assessed by immunohistochemistry) and achieved the same Th2-like anti-inflammatory cytokine response (240).

Fibrinogen-derived citrullinated peptides have been intensively investigated due to their high capacity to scavenge ACPA isolated from RA patients, showing that cyclized structures bind with higher affinities (247, 248). A fibrinogen-derived citrullinated cyclic peptide have also been reported to treat CIA rat, showing a significant decrease of joint swelling when compared to untreated or non-citrullinated peptide control groups, along with an increase of IL-10 (241). Data obtained from McElwee et al. suggests that citrullinated fibrinogen may have potent tolerogenic properties (242). When they immunized a transgenic HLA-DR mice model with citrullinated peptides derived from cartilage intermediate layer protein or fibrinogen, the arthritis-initiating response from CD4^+^ T cells upon presentation of citrullinated antigens was not observed. Instead, expansion of CD4^+^ T cell population binding to these HLA-DR:citrullinated peptides was observed, with lower levels of Th1 and higher levels of Treg cells. These results were not observed when same mice model was immunized with citrullinated vimentin or enolase 1 peptides (242). Restoration of Treg over Th or Teff cell populations and balance recovery was also seen in the already discussed Gertel et al. study, where the multiepitope used to treat adjuvant-induced RA rats contained citrullinated fibrinogen (239); or on the successful Rheumavax trial, which contained tolDCs introduced to citrullinated fibrinogen, among other citrullinated peptides (195).

### 6.4 Modulating ACPAs

Gomez et al. recently showed that the injection of several ACPAs isolated from RA patients ameliorated inflammation and disease severity in collagen antibody-induced arthritis (CAIA) model (127) adding up to a long list of examples where ACPAs had therapeutic or preventive effects in RA murine models (243, 244, 249). It is worth mentioning that in the experiments carried out by Gomez et al, patients derived ACPAs were grouped and dosed based on the predominant citrullinated antigen they targeted and all groups had similar effects specially when injecting in early steps of CAIA (127).

**Figure 4:**
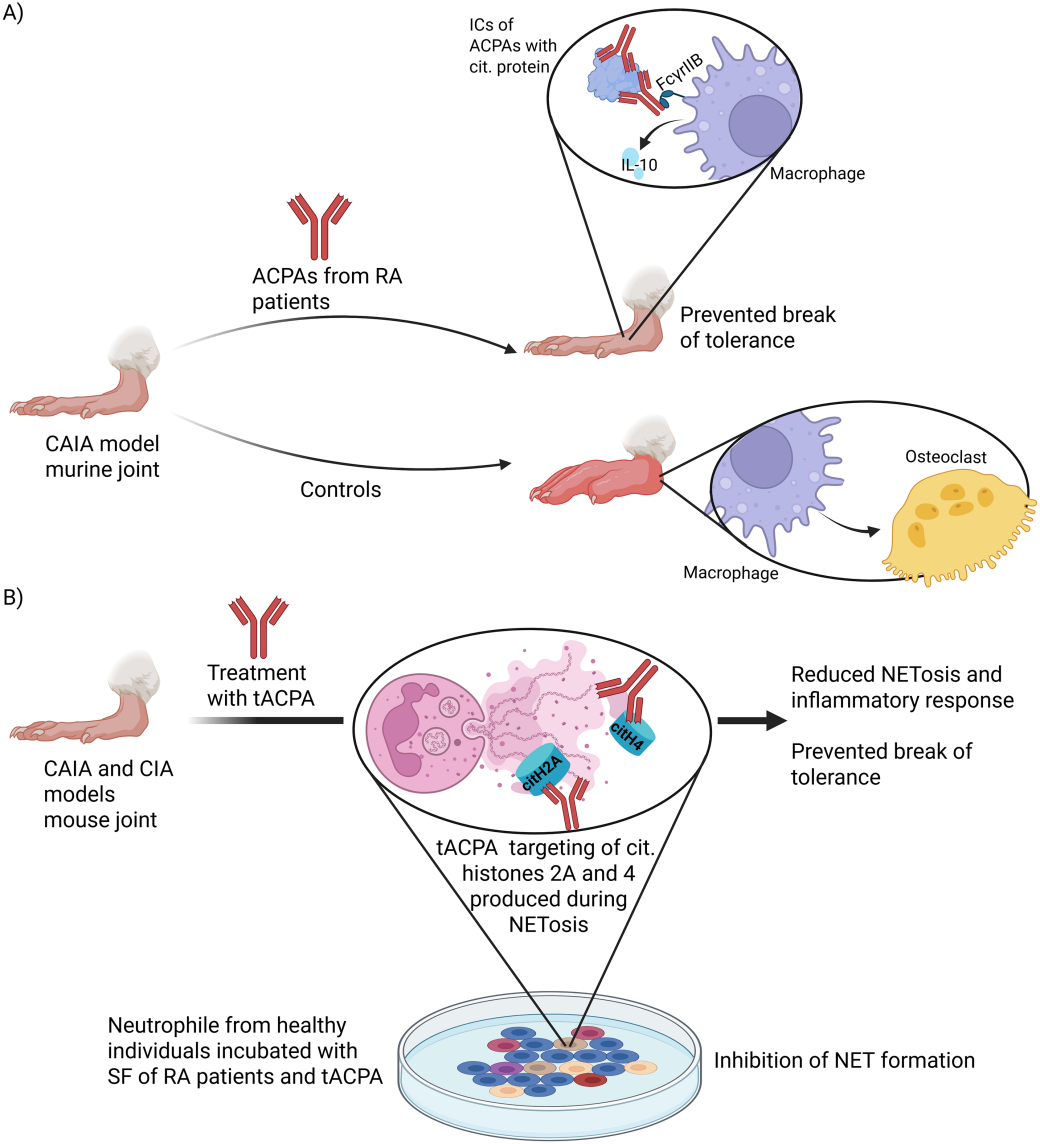
Role of ACPAs in prevention of tolerance breach in murine RA models. **(A)** Injection of ACPAs isolated from RA patients prevented inflammation and disease progression in CAIA model (127, 243). ACPAs were shown to form ICs with citrullinated proteins and interact with FcγRs IIB from osteoclasts, promoting IL-10 release and reducing their osteoclastogenesis (136, 243). **(B)** Treatment with tACPA targeting citrullinated histones citH2A and citH4 prevented the development of inflammatory response in CAIA mice and inhibited NETosis in both murine model and human neutrophile stimulated with SF from RA patients and other disease stimuli. IC, Immune Complex; tACPA, therapeutic ACPA; cit., citrullinated (245). Created with BioRender.

This seems to point out that in a target independent manner, ACPAs have the ability to induce tolerance (in earlier stages) or prevent break of tolerance that will exacerbate the disease in a CAIA model (**Figure 4A**). He et al. also injected patient derived ACPAs in healthy mice to observe neither arthritogenicity nor pain signs. One of the antibodies protected the mice from antibody-induced arthritis (CAIA model) by forming ICs with citrullinated α-enolase and other few citrullinated proteins from SF which posteriorly bound to macrophages in the joints, resulting in increased secretion of anti-inflammatory IL-10 and reduced osteoclastogenesis (243). Authors attributed the reduction of osteoclastogenesis to the interaction of the interaction of these ICs with FcγRIIB in macrophages (**Figure 4A**) (136, 243). Another example of ACPA usage to prevent the development of inflammation not only in CAIA RA model but also in other NET-mediated pathologies as inflammatory bowel disease, pulmonary fibrosis and sepsis, was reported by Chirivi et al. (245) They developed an ACPA that specifically targets citrullinated histones citH2A and citH4, which are generated during NET release. Their lead therapeutic ACPA prevented the inflammatory response in several autoimmune models (incl. CAIA), reduced inflammation in CIA mice RA model, inhibited NET formation in both murine models and *in vitro* experiments where healthy individuals neutrophiles were stimulated with different disease-related stimuli (including SF containing ACPAs from RA patients) and potentially favoured macrophage mediated clearance of NETs (**Figure 4B**) (245).

## 7 Conclusions and future perspectives

Restoring immune tolerance in RA marks a meaningful transition in therapeutic strategies, focusing on re-educating the immune system rather than just suppressing its activity. Promising preclinical and early clinical evidence supports this approach, indicating that targeting the underlaying mechanisms of autoimmunity may provide durable disease control with reduced systemic immunosupresion.

Peptide-based immunotherapies, such as Jusvinza, DNAJP1, Rheumavax and DEN-181 have demonstrated the capacity to modulate key immune pathways, Treg populations, lowering Th1/Th17-driven inflammation, and, in some cases, reducing circulating ACPA levels. Parallelly, tolerogenic cell therapies and monoclonal antibodies targeting co-stimulatory pathways (e.g., abatacept, peresolimab, dazodalibep) further highlight the feasibility of antigen-specific or pathway-guided tolerance induction. Collectively, these advances mark a gradual yet meaningful transition toward tolerogenic disease-modifying strategies.

Among these emerging modalities, peptide-based therapies offer several advantages over extensively used monoclonal antibodies. Tolerogenic peptides engage with autoreactive T and B cells with high specificity while being synthetically accessible, structurally well defined, and easier to rationalize for epitope optimization. Compared to monoclonal antibodies or binding proteins, their lower production costs and stability further enhance their suitability for long-term or preventive use.

Dose requirements also illustrate peptides efficiencies for the described preliminary and exploratory trials. As summarized in **Table 1**, DEN-181 achieved immunomodulatory effects at only 126 µg of CII peptide in a single dose, and Jusvinza demonstrated clinical tolerogenic benefits with 5 doses of 5 mg, both markedly lower than conventional csDMARDs (e.g., ≥7.5 mg/week for methotrexate or 400 mg/day for hydroxychloroquine, both according to FDA label) and substantially below the 2 g required for B-cell depletion with rituximab.

Administration routes constitute another advantage of peptide-based tolerogenic approaches. Subcutaneous formulations, as shown for DEN-181, Jusvinza and CPEP02 (in preclinical development), successfully induced immune modulation, while oral administration of DNAJP1 achieved significant T-cell phenotypic and cytokine profile shifts. Although daily dosing over six months was required, the total 4.5 g peptide exposure remains feasible considering the production scales of peptides compared to those of monoclonal antibodies. The DNAJP1 trials thus represent a key milestone in advancing orally delivered tolerogenic immunotherapies, with future peptide design optimization likely to improve dosage efficiency and patient compliance.

Despite these advances, achieving long-term immune tolerance requires a deeper understanding of the immune regulation, ACPA biology and the precise mechanisms that drive autoimmunity in RA. The absence of a prevalent murine model used in preclinical research (**Table 2**), complicates preclinical translation. Therefore, quantitative joint swelling measurements (paw diameter) and harmonized disease scoring systems should be adopted as standardized endpoints to facilitate cross-study comparison. Splenic or synovial cell profiling, which is not carried out in most preclinical studies described, is essential to elucidate the tolerogenic effects of these treatments in immune cell differentiation and regulation.

Future research must address these gaps through systems-level analyses integrating multi-omics, single-cell immunophenotyping, and spatial transcriptomics to map the dynamics of tolerance restoration *in vivo*. Such efforts will be critical to distinguish true immune reprogramming from transient immunomodulation.

In summary, the combination of peptide-based therapies with conventional DMARDs or targeted biologics also represents a promising therapeutic framework to enhance efficacy and maintain remission while minimizing systemic immunosuppression, reducing the risk of relapsing and improving the quality of life for patients with RA.
